# Examining the Role of Attention Deficits in the Social Problems and Withdrawn Behavior of Children With Sluggish Cognitive Tempo Symptoms

**DOI:** 10.3389/fpsyt.2021.585589

**Published:** 2021-05-04

**Authors:** Trevor W. K. Yung, Cynthia Y. Y. Lai, Jacob Y. C. Chan, Shamay S. M. Ng, Chetwyn C. H. Chan

**Affiliations:** ^1^Department of Rehabilitation Sciences, The Hong Kong Polytechnic University, Kowloon, Hong Kong; ^2^Applied Cognitive Neuroscience Laboratory, Department of Rehabilitation Sciences, The Hong Kong Polytechnic University, Kowloon, Hong Kong; ^3^University Research Facility in Behavioral and Systems Neuroscience, The Hong Kong Polytechnic University, Kowloon, Hong Kong; ^4^Department of Counseling Psychology, Social Psychology, and Counseling, Ball State University, Muncie, IN, United States

**Keywords:** children, sustained attention, selective attention, social problems, sluggish cognitive tempo

## Abstract

Previous studies have found that sluggish cognitive tempo (SCT) symptoms are often associated with social problems and withdrawn behavior. However, the possible neuropsychological mechanism underlying this relationship remains unclear. Some studies have also found that SCT symptoms are related to deficits in sustained attention and selective attention. However, no study has examined whether attention deficits are related to social problems and withdrawn behavior in children with SCT. This study was the first to examine the neuropsychological correlates of social problems and withdrawn behavior among children with SCT symptoms. The results showed that sustained attention measure (omission) predicted the severity of social problems and withdrawn behavior in children with SCT even after controlling for symptoms of attention-deficit hyperactivity disorder. Selective attention measure (response latency mean) was also found to predict the severity of social problems. These results suggest that the social problems commonly exhibited by children with SCT are related to deficits in sustained attention and attentional control. Thus, our results provide an initial support to the link between attention deficits and social problems among children with SCT.

## Introduction

Sluggish cognitive tempo (SCT) is an attention problem characterized by a cluster of symptoms including difficulties in sustained alertness (e.g., daydreaming) and slowness in thoughts and actions (e.g., drowsiness and delayed responsiveness) (1–3). These symptoms, which manifest in both children and adults, are often associated with impairments in daily functioning, such as emotional difficulties (e.g., anxiety and depression), social problems, and academic learning difficulties (1, 4). Although recent findings have shown that SCT is distinct from any type of attention-deficit hyperactivity disorder (ADHD) (5–8), its underlying neuropsychological and neurophysiological mechanisms are not yet clearly understood.

### SCT and Social Problems

Among different clinical features of SCT, social problems have attracted much attention from researchers in this field. Social problems refer to two core difficulties: social rejection and difficulties in initiating or maintaining social relationships with others (9). Many previous studies have found associations between social problems and SCT symptoms after controlling for the influence of ADHD symptoms on the severity of social problems (10–13).

Willcutt et al. (7) found that SCT symptoms, not DSM-IV inattention symptoms, were associated with social isolation, but found no association between SCT symptoms and the tendency to be disliked by peers. This implied that the nature of social problems in children with SCT is related to their withdrawal from social interaction rather than their being rejected by their peers. Sáez et al. (14) also found that child-rated SCT symptoms were associated with mother-rated social impairment and that SCT was associated with both loneliness and a preference for solitude among these children.

In a recent study by Becker et al. (15), the nature of social problems was found to differ between children with high SCT symptoms and those with ADHD. Consistent with other studies, the authors found that SCT symptoms were uniquely associated with social behaviors characterized by passive/low social engagement, withdrawal, and isolation. In contrast, ADHD hyperactivity symptoms were significantly associated with other aspects of social functioning characterized by improper responding, active peer exclusion/rejection, and poor self-control in social situations, whereas ADHD inattention symptoms were not associated with social impairments.

Becker (1) conducted a longitudinal study to examine whether SCT symptoms would predict poorer social functioning over a 6-month period in a sample of elementary school students while controlling for baseline social functioning, ADHD, oppositional defiant disorder (ODD), and internalizing symptoms. The results of this teacher-rated study indicated that SCT was a strong predictor of children's poorer later peer functioning across domains of popularity, social preference, and peer relations (10). These results supported the findings of previous studies that SCT symptoms are strongly associated with the development of social difficulties.

Social difficulties in children with SCT are reflected not only in behavioral report measures but also in laboratory findings. A previous study using a computerized chat room task found that SCT symptoms alone predicted fewer responses, a reduced ability to attend to subtle social cues, a weaker memory for conversations, and a smaller proportion of hostile responses compared to normal control (16).

In summary, passive social engagement, withdrawal, and isolation are the unique characteristics of children with high SCT symptoms, in contrast to the social deficits observed in children with ADHD. However, it is unclear why these children manifest such tendencies in social functioning and which neuropsychological factors are involved in the development of such social difficulties.

### Attention Deficits Associated With Social Problems

Despite the establishment of a clear link between social problems and SCT symptoms, the possible neuropsychological mechanism that contributes to the social problems exhibited by children with high SCT symptoms remains unclear. Understanding this mechanism is important because it may improve our understanding of the underlying deficits that likely contribute to the common problems associated with SCT. Consequently, such an understanding would inform the development of a more comprehensive model to explain SCT. Andrade et al. (17) proposed that cognitive processes, such as attention, affect children's social functioning. They suggested two types of attention that may be related to social problems in children: sustained attention and selective attention (17). Sustained attention may affect children's ability to maintain focus during conversations and social play and attend to important social cues during social interaction (17). Children who fail to maintain attention during social interaction may be seen by their peers as disinterested in such interaction. Subsequently, their peers may not approach them for further social interaction (17). The second type of attention, selective attention, may be related to social problems. In the social world, it is important for children to shift their focus to relevant social cues and to inhibit non-relevant cues so that they can make appropriate decisions and display appropriate behaviors (17). In a study by Andrade et al. (17), a continuous performance test (CPT) was performed in 101 children aged 6–12 months, comprising children with formally diagnosed ADHD, children who were referred for an assessment of attention difficulties but were not diagnosed with ADHD, and children with typical development. The results showed that the percentage of correct responses in 2- and 4-s interstimulus intervals (ISIs) was significantly and negatively associated with social problems after controlling for the hyperactivity score. Fewer correct responses in longer ISIs were associated with more social problems, possibly because longer ISIs require the subjects to be more vigilant during the task. Thus, these results suggested a link between sustained attention deficit and social problems among children. A sustained attention deficit may negatively affect a child's ability to acquire appropriate social information during conversations and social play with their peers and subsequently decrease their initiative to interact with others (17). Thus, sustained attention and selective attention were concluded to be related to social difficulties.

### SCT and Neuropsychological Deficits

Researchers have examined whether deficits in executive functioning could account for SCT symptoms. In a study comprising 209 children recruited from the community sample, Wåhlstedt and Bohlin (18) found that ADHD inattention symptoms were independently associated with performance in inhibitory control, working memory, and reaction time variability, whereas SCT symptoms were associated with performance in sustained attention alone. In another study, Willcutt et al. (7) recruited 410 individuals with DSM-IV ADHD and 311 healthy individuals as the comparison group from the community and found that only sustained attention deficits remained significantly associated with SCT symptoms after controlling for inattention and hyperactivity/impulsivity symptoms. They also did not find any significant relationship between the severity of SCT symptoms and performance in executive functioning tasks (e.g., response inhibition, working memory, naming speed, and response variability). Baytunca et al. (19) compared the SCT-and-ADHD group (*n* = 42) with the ADHD-only group (*n* = 41) and the control group (*n* = 24) in terms of performance on the CNS Vital Signs Battery. Their results revealed that the SCT-ADHD group performed significantly worse on the CPT than did the ADHD-only and control groups, indicating that the SCT-ADHD group had a poorer ability to sustain attention than did the ADHD-only group. The performance of the SCT-ADHD group did not decline in any other neuropsychological domain, such as processing speed, visual/auditory memory, psychomotor speed, and reaction time, compared with the other two groups. Bauermeister et al. (20) recruited a community sample of 140 children aged 6–11 years and found no significant correlations between SCT and executive functioning (including working memory, processing speed, memory retrieval, interference control, and planning/problem solving). All of these reports suggest that the severity of SCT is not associated with executive functioning deficits that have been shown to be related to ADHD. Rather, SCT could be more related to sustained attention deficits reflected by laboratory attention tests (e.g., the Go/No-go test). Therefore, it is more likely that difficulties in sustained attention, rather than in executive functioning, are a core deficit associated with SCT.

Besides sustained attention, difficulties in selective attention have been suggested to be another possible neuropsychological deficit in SCT. Huang-Pollock et al. (21) compared the performance of children aged 8–12 years with or without SCT symptoms on the selective attention paradigm. They found that children with SCT had significantly higher response time interference than did children without SCT when more stimuli were presented on the computer screen (6-stimulus condition). Based on this result, Huang-Pollock et al. concluded that children with SCT may have abnormal early selective attention processing. Baytunca et al. (19) also found that the ADHD group with SCT symptoms had fewer correct responses and more errors on the shifting attention test than did the ADHD-only group. All of these findings suggest that SCT symptoms are related to selective attention deficits.

Sustained attention and selective attention are crucial to children's success in social interaction with others because they enable children to continuously attend to the social cues and to select appropriate social information based on which to provide appropriate social responses (17). The withdrawn behavior and social problems of children with SCT may be due to their inability to sustain attention and select appropriate social information during interactions. However, no study has yet examined this hypothesis.

### Aim and Hypotheses

This study aimed to examine the associations of deficits in sustained attention and selective attention with social problems in school-aged children with SCT symptoms and to characterize these associations by exploring the specific parameters of these attention measures that contribute to the problems.

In this study, sustained attention and selective attention deficits were conceptualized to contribute to the social problems of children with SCT in two ways: (a) sustained attention deficits hamper the ability of these children to be alert to social information during social play, and (b) selective attention deficits hamper the ability of these children to select appropriate social information based on which to formulate social responses. Therefore, we hypothesized the following:

1. Sustained attention measures are significantly associated with the severity of social problems and withdrawn behavior in children with high SCT symptoms, as measured by a parent rating scale; and
2. Selective attention measures are also significantly associated with the severity of social problems and withdrawn behavior in children with high SCT symptoms, as measured by a parent rating scale.

## Methods

### Participants

The participants recruited in this study were required to meet the following inclusion criteria: (a) aged 6–12 years and (b) a full-scale IQ score ≥ 80. Potential participants who met any of the following criteria were excluded: (a) any psychiatric diagnosis, including autism, ODD, and conduct disorder or (b) a T-score > 60 in the Chinese version of the Strength and Weaknesses of ADHD Symptoms and Normal Behavior scale [SWAN; (22, 23)]. Participants with other psychiatric disorders [e.g., ADHD, childhood autism, or ODD/conduct disorder] were excluded because these disorders have been found to be associated with different neuropsychological deficits and could have therefore confounded the relationship between attention deficits and social problems in our study.

Ethical approval was obtained from the University Ethics Committee before beginning the study (Reference number: HSEARS20150724002-03). Eighty-eight primary school students aged 6–12 years were recruited by a convenience sampling method. Electronic posters were sent to all parents of primary school students and they were asked to send the posters to other parents who may have interest to join. Parents who were interested in participating in this research were asked to contact the researchers by phone. The researchers briefly explained the aim and procedure of the study to the parents over the phone call. If the parents were interested in the study, they were invited to schedule a time with the examiner to undergo the study tests. On the day of testing, the researchers gave a research package (information on the nature and purpose of the study and a written consent form which informed them they could withdraw from the study anytime and their personal details would not be disclosed to other people who were not in the research team) and re-explained the details of the research to the parents. Signed informed consent forms were collected prior to data collection.

Among the participants, 40 children (*M*_age_ = 114.08 months, *SD* = 18.806 months; 52.5% female) were grouped into the high SCT group while 48 children (*M*_age_ = 108.92 months, *SD* = 20.739 months; 45.8% female) were grouped into the normal control group using the median-split method on the scores of the SCT-rating scale (median cut-off value = 25). No significant difference was observed in children's age between the two groups, *t*_(76)_ = 1.717, *p* = 0.90.

### Procedure and Experimental Setup

Each participant was asked to attend one 1.5-h testing session conducted in a laboratory free from visual or auditory distractions. Breaks were offered before or during the tests.

#### Measurement Instruments

Each participant underwent the following neuropsychological tests:

*Wechsler Intelligence Scale for Children, Fourth Edition–Hong Kong (Short-Form)* [*WISC-IV-HK-SF*; (24)]. This is a short version of the WISC-IV, a formal intelligence scale. It comprises four subscales: similarities, matrix reasoning, coding, and digit span. The raw scores for these four subscales are combined to give a total raw score, which is then transformed into a full-scale IQ score.

*Child Behavior Checklist for Ages 6–18* [*CBCL/6-18;* (25)]. The CBCL/6-18 is a parent rating scale that measures the emotional and behavioral problems of children aged 6–18 years. Each item is rated on a 3-point scale (0 = *not true*, 1 = *somewhat or sometimes true*, 2 = *very true or often true*). The CBCL comprises eight scales: anxiety/depression, somatic complaints, social problems, thought problems, attention problems, rule-breaking behavior, and aggressive behavior. The raw scores for each scale are combined to give a total raw score, which is then transformed into a standardized subscale score. The CBCL has been reported to have good to excellent test-retest reliability (intra-class correlation coefficient:0.66–0.87) and good discriminant validity (26).

*SWAN–Chinese Version*. The SWAN is a parent and teacher rating scale used to screen for ADHD symptoms (23). The translated Chinese version used in this study has been validated for use in children in Hong Kong (22). The scale comprises 18 items that measure a child's control of their attention, impulses, and activity. The items are divided into two subscales of nine items each, which address inattention and hyperactivity/impulsivity. The total and subscale scores are generated by summing the raw item scores, which can be expressed as T-scores. A higher score indicates fewer ADHD symptoms. In this study, only the total SWAN scores were used. All of the total and subscale scores for both the parent and teacher versions of SWAN have been found to have very good internal consistency (alpha >0.9) (22) and good discriminant validity (AUC >0.8) (22).

*SCT Scale* (3). This 14-item parent and teacher rating scale is used to measure SCT symptoms in children. Each item is rated on a 7-point scale (0 = *not at all* to 6 = *very much*). Similar to previous studies that used this scale (15, 27), 10 items were selected for use instead of the full scale in the present study. These 10 items were found to yield consistent loadings on the SCT factor, but not on the ADHD inattention factor (5). This unified 10-item construct was consistent with the participants' characteristics and the hypotheses of the study. The internal consistency of the 14-item SCT scale is high when both parent (alpha >0.8) and teacher (alpha >0.9) ratings are measured (3). Additionally, the 10-item version yielded a high level of internal consistency in the present study (Cronbach's alpha = 0.898).

*CANTAB Attention Switching Task* [*AST;* (28)]. The AST is a measure of cued attentional shifting task in which an arrow appears on the right- or left-hand side of a computer screen and the subject is required to provide a right or left response based on the cues that indicate whether they have to respond according to the direction of the arrow or according to the side of the screen on which the arrow appears. There are three blocks in this task: direction block, side block, and switching block. In the direction block, subjects are asked to provide a right or left response according to the direction of the arrow on the computer screen. In the side block, subjects are asked to respond according to the side of the screen on which the arrow appears. In the switching block, subjects are required to respond according to either cue (direction or side). The total number of correct responses across three blocks (AST total correct) and the mean of the response times in three blocks (AST reaction latency [mean]) were used in the present study.

*Cued Continued Performance Test* [*CCPT;* (29)]. The CCPT was used to measure the performance of sustained attention ability in the present study. The subjects were required to press the response button when the target stimuli appeared on the computer screen, while required to withhold from responding when the non-target stimuli appeared. Stimuli were letters presented at the center of the computer screen (one letter at a time for 200 ms with an ISI of 1,650 ms) in a pseudo-randomized order. Target stimuli were a paired sequence of stimuli in which the letter O was presented first, followed by the letter X. In the sequence of non-target stimuli, the letter O was presented first, followed by non-X letters. The stimulus set consisted of two blocks of 200 trials, and the subjects were asked to take a 1-min break after the first block to avoid mental fatigue. In each block, 40 stimuli were “O letter” stimuli, 20 were target stimuli, and 20 were non-target stimuli. The remaining 120 stimuli were distractor letters (letters other than O or an X without a preceding O). The measures generated from each block of the tests were the numbers of target hits (X with a preceding O), omission errors (no response toward the target), commission errors (response to non-target and distractor stimuli), and response latency toward the target. The final measures used in the analysis in the current study were calculated by deducting the value of each measure in block 1 from the value of that measure in block 2 (i.e., the final measure of omission errors = the number of omission errors in block 2—the number of omission errors in block 1). The rationale behind such calculation was to obtain a measurement of performance deterioration over time, which was assumed to reflect sustained attention (18).

### Data Analysis

A multivariate analysis of variance (MANOVA) was used to examine the behavioral difference (reflected by the CBCL) between the high SCT group and the control group. Eight subscale scores (Withdrawn, Somatic Complaints, Anxious/Depressed, Social Problems, Thought Problems, Attention Problems, Delinquent Behavior, and Aggressive Behavior) of the CBCL were used as dependent variables. Subsequently, a bivariate correlation analysis was performed to examine the correlations between the CBCL Social Problem/Withdrawn subscale scores and the AST scores (AST total correct and AST reaction latency [mean]) and between the CBCL Social Problem/Withdrawn subscale scores and the CCPT scores (correct responses, omission errors, commission errors, and response time). The AST scores and CCPT scores were found to have significant associations with the CBCL Social Problem subscale score and/or the CBCL Withdrawn subscale score; thus, these scores were entered into the regression analysis as independent variables. Significant correlations were observed between CCPT scores (*r* = −*0.3*60 to 0.386) and between AST scores (*r* = 0.442). Therefore, to minimize multicollinearity, a stepwise regression was performed to examine the contributions of the different AST scores/CCPT scores to the severity of social problems/withdrawn behavior. All of these analyses were conducted using SPSS 23.0 (IBM, U.S.A.), and the significance level was set at 0.05.

## Results

### Behavioral Difference Between the High SCT Group and the Control Group

A one-way MANOVA was conducted with the SCT group status (high SCT vs. control) as the independent variable and the CBCL subscale scores (Withdrawn, Somatic Complaints, Anxious/Depressed, Social Problems, Thought Problems, Attention Problems, Delinquent Behavior, and Aggressive Behavior) as the dependent variables. A higher score in the CBCL subscales represents more clinical problems in daily life.

Significant differences were found in the CBCL subscale scores between the high SCT and control groups, *F*_(8, 75)_ = 3.617, *p* < 0.05; Wilk's Lambda = 0.722, partial eta squared = 0.278. After Bonferroni correction (*p* = *0.0*5/8 = 0.00625), the high SCT group showed significantly higher scores than the control group on the Withdrawn subscale, *F*_(1, 82)_ = 16.829, *p* < 0.006; partial eta squared = 0.170, the Anxious/Depressed subscale, *F*_(1, 82)_ = 10.709, *p* < 0.006; partial eta squared = 0.116, the Social Problems subscale, *F*_(1, 82)_ = 18.166, *p* < 0.006; partial eta squared = 0.181, and the Attention Problems subscale, *F*_(1, 82)_ = 14.564, *p* = 0.006; partial eta squared = 0.151 (see Table 1). These results suggest that the high SCT group had more withdrawn behavior, anxiety/depression, and attention and social problems than did the control group.

**Table 1:** Mean and standard deviation of the CBCL subscale scores in the SCT and control groups.

	**SCT group**	**Control** **group**	***F***	***p***	**Partial eta** **squared**
	***M* (*SD*)**	***M* (*SD*)**			
Withdrawn	3.63 (3.267)	1.33 (1.79)	16.829	0.000	0.170
Somatic complaints	1.47 (1.751)	1.15 (1.813)	0.675	0.414	0.008
Anxious/Depressed	4.68 (3.655)	2.24 (3.192)	10.709	0.002	0.116
Social problems	3.82 (2.103)	2.00 (1.801)	18.166	0.000	0.181
Thought problems	0.84 (1.151)	0.37 (0.741)	5.165	0.026	0.059
Attention problems	5.79 (2.961)	3.43 (2.689)	14.564	0.000	0.151
Delinquent behavior	2.50 (1.885)	1.50 (1.847)	5.987	0.017	0.068
Aggressive behavior	8.03 (6.441)	6.15 (5.432)	2.094	0.152	0.025

*CBCL, Child behavior checklist; SCT, Sluggish cognitive tempo; SD, Standard deviation*.

### Bivariate Associations Between Sustained Attention Measures (CCPT) and the CBCL Social Problem and Withdrawn Subscale Scores

A bivariate correlation analysis was used to test the associations of the sustained attention measures with the subjects' CBCL Social Problem and Withdrawn subscale scores in both the high SCT and control groups. The means and standard deviations of the CCPT measures, the Social Problem subscale score, and the Withdrawn subscale score of both groups are shown in Table 2. In the high SCT group, significant correlations were found between the Withdrawn subscale score and CCPT omission and commission and between the Social Problem subscale score and CCPT omission (Table 3). In the control group, significant correlations were found between the Withdrawn subscale score and CCPT commission and between the Social Problem subscale score and CCPT commission (Table 4).

**Table 2:** Mean and standard deviation of CCPT measures in the SCT and control groups.

	**SCT group**	**Control group**
	***M* (*SD*)**	***M* (*SD*)**
Correct response	−0.4872 (2.316)	−0.9149 (2.04)
Omission	0.9487 (2.743)	0.9149 (2.041)
Commission	−1.615 (25.31)	1.702 (10.24)
Response time	48.85 (116.76)	53.68 (96.83)

*CCPT, Cued continued performance test; SCT, Sluggish cognitive tempo; SD, Standard deviation*.

**Table 3:** Correlation coefficients of the CCPT measures with the CBCL withdrawn and social problem subscale scores in the SCT group.

	**CCPT measures**			
	**Correct**	**Omission**	**Commission**	**Response** **time**
Withdrawn	0.040	0.539^**^	0.406^*^	0.041
Social problem	−0.105	0.432^**^	0.167	0.136

*CCPT, Cued continued performance test; CBCL, Child behavior checklist; SCT, Sluggish cognitive tempo; Withdrawn, CBCL Withdrawn subscale score; Social problem, CBCL Social Problem subscale score; Correct, CCPT correct response; Omission, CCPT omission errors; Commission, CCPT commission errors; Response time, CCPT response time*.

* *p < 0.05*,

** *p < 0.01*.

**Table 4:** Correlation coefficients of the CCPT measures with the CBCL withdrawn and social problem subscale score in the control group.

	**CCPT measures**			
	**Correct**	**Omission**	**Commission**	**Response** **time**
Withdrawn	0.039	−0.039	0.405^**^	−0.173
Social problem	0.065	−0.065	0.340	−0.258

*CCPT, Cued continued performance test; CBCL, Child behavior checklist; Withdrawn, CBCL Withdrawn subscale score; Social problem, CBCL Social Problem subscale score; Correct, CCPT correct response; Omission, CCPT omission errors; Commission, CCPT commission errors; Response time, CCPT response time*.

** *p < 0.01*.

### Bivariate Associations Between Attention Shifting Measures (AST) and the CBCL Social Problem and Withdrawn Subscale Scores

A bivariate correlation analysis was used to test the associations between the AST measures and the CBCL Social Problem and Withdrawn subscale scores. The means and standard deviations of the AST measures in both the high SCT and control groups are shown in Table 5. In the high SCT group, significant correlations were found between the Withdrawn subscale score and the AST total correct, between the Social Problem subscale score and the AST total correct, and between the Social Problem subscale score and the AST response latency (mean) (Table 5). In the control group, no significant correlation was found between any AST measure and the CBCL Social Problem and Withdrawn subscale scores (Table 6).

**Table 5:** Mean and standard deviation of the AST measures in the SCT and control groups.

	**SCT group**	**Control group**
	***M* (*SD*)**	***M* (*SD*)**
Total correct Response	135.64 (27.2)	137.36 (17.7)
latency (mean)	732.66 (164.5)	756.77 (123)

*AST, Attention switching task; SCT, Sluggish cognitive tempo; SD, Standard deviation*.

**Table 6:** Correlation coefficients of the AST measures with the CBCL withdrawn and social problem subscale scores in the SCT group.

	**AST measures**	
	**Total correct**	**Reaction latency (mean)**
Withdrawn	−0.320^*^	−0.070
Social problem	−0.359^*^	−0.414^**^

*AST, Attention switching task; CBCL, Child behavior checklist; SCT, Sluggish cognitive tempo; Withdrawn, CBCL Withdrawn subscale score; Social problem, CBCL Social Problem subscale score; Total correct, AST total correct response; Reaction latency (mean), mean of the AST reaction latency.*

* *p < 0.05*,

** *p < 0.01*.

### Use of CCPT Measures to Explain the CBCL Withdrawn and Social Problem Subscale Scores

A stepwise regression was performed to examine the associations between the CCPT measures and the CBCL Withdrawn and Social Problem subscale scores after entering the SWAN ADHD score into the model to control for ADHD symptoms (Step 1). Subsequently, the CCPT measures (omission and commission) were entered using the stepwise method (entry probability = 0.05; removal probability = 0.10) (Step 2) to explain the variance of the CBCL Withdrawn and Social Problem subscale scores in both groups. To avoid type 1 error, Bonferroni correction was used to adjust the *p*-value. The adjusted *p*-value was 0.0125 (0.05/4).

In the high SCT group, the SWAN ADHD score did not contribute significantly to the regression model to explain the CBCL Withdrawn subscale score, *F*_(1, 37)_ = 1.425, *p* > 0.0125 (Table 7) or the CBCL Social Problem subscale score, *F*_(1, 37)_ = 0.440, *p* > 0.0125 (Table 8). The stepwise analysis identified only CCPT omission as a significant independent variable in the regression model to explain the CBCL Withdrawn and Social Problem subscale scores (Tables 8, 9). The CCPT omission measure explained 25.8% of the variance in the CBCL Withdrawn subscale score, *F*_(1, 36)_ = 13.174, *p* < 0.0125, and 17.5% of the variance in the CBCL Social Problem subscale score, *F*_(1, 36)_ = 7.75, *p* < 0.0125.

**Table 7:** Correlation coefficients of the AST measures with the CBCL withdrawn and social problem subscale scores in the control group.

	**AST measures**	
	**Total correct**	**Reaction latency (mean)**
Withdrawn	−0.282	0.044
Social problem	−0.128	−0.030

*AST, Attention switching task; CBCL, Child behavior checklist; Withdrawn, CBCL Withdrawn subscale score; Social problem, CBCL Social Problem subscale score; Total correct, AST total correct response, Reaction latency (mean) = mean of the AST reaction latency*.

**Table 8:** Regression model of the CCPT measures as the predictors of the CBCL withdrawn subscale score in the SCT group.

**Variable**	**Unstandardized B**	**Coefficient** **standard error**	**Standardized** **coefficient beta**	***t***	**Sig**.	***R***	***R*^**2**^**	**Adjusted *R*^**2**^**	***R*^**2**^ change**
**Step 1**									
SWAN ADHD score	−0.059	0.049	−0.193	−1.19	0.24	0.193	0.037	0.011	0.037
**Step 2**									
SWAN ADHD score	−0.022	0.044	−0.070	−0.488	0.628	0.543	0.295	0.256	0.258^**^
Omission	0.608	0.167	0.522	3.63	0.001				

*CCPT, Cued continued performance test; CBCL, Child behavior checklist; SCT, Sluggish cognitive tempo; ADHD, Attention-deficit hyperactivity disorder; SWAN, the Strength and Weaknesses of ADHD Symptoms and Normal Behavior scale; Omission, CCPT omission errors*.

** *p < 0.01*.

**Table 9:** Regression model of the CCPT measures as the predictors of the CBCL social problem subscale score in the SCT group.

**Variable**	**Unstandardized B**	**Coefficient** **standard error**	**Standardized** **coefficient beta**	***t***	**Sig**.	***R***	***R*^**2**^**	**Adjusted *R*^**2**^**	***R*^**2**^ change**
**Step 1**									
SWAN ADHD score	−0.022	0.033	−0.108	−0.664	0.511	0.108	0.012	−0.015	0.012
**Step 2**									
SWAN ADHD score	−0.002	0.031	−0.008	−0.050	0.960	0.432	0.187	0.142	0.175^**^
Omission	0.326	0.117	0.430	2.784	0.009				

*CCPT, Cued continued performance test; CBCL, Child behavior checklist; SCT, Sluggish cognitive tempo; ADHD, Attention-deficit hyperactivity disorder; SWAN, the Strength and Weaknesses of ADHD Symptoms and Normal Behavior scale; Omission, CCPT omission errors.*

** *p < 0.01*.

In the control group as well, the SWAN ADHD score did not contribute significantly to the regression model to explain the CBCL Withdrawn subscale score but contributed significantly to explain the CBCL Social Problem subscale score (Tables 10, 11). CCPT commission was identified as a significant independent variable that could explain the CBCL Withdrawn subscale score. It explained 15.3% of the variance of the CBCL Withdrawn subscale score, *F*_(1, 44)_ = 8.208, *p* < 0.0125. The CCPT commission measure was not a significant independent variable to explain the CBCL Social Problem subscale score, *F*_(1, 44)_ = 5.557, *p* > 0.0125.

**Table 10:** Regression model of the CCPT measures as the predictors of the CBCL withdrawn subscale score in the control group.

**Variable**	**Unstandardized B**	**Coefficient** **standard error**	**Standardized** **coefficient beta**	***t***	**Sig**.	***R***	***R*^**2**^**	**Adjusted *R*^**2**^**	***R*^**2**^ change**
**Step 1**									
SWAN ADHD score	−0.023	0.021	−0.161	−1.095	0.279	0.161	0.026	0.004	0.026
**Step 2**									
SWAN ADHD score	−0.018	0.020	−0.122	−0.889	0.379	0.423	0.179	0.142	0.153^**^
Commission	0.068	0.024	0.393	2.865	0.006				

*CCPT, Cued continued performance test; CBCL, Child behavior checklist; ADHD, Attention-deficit hyperactivity disorder; SWAN, the Strength and Weaknesses of ADHD Symptoms and Normal Behavior scale; Commission, CCPT commission errors*.

** *p < 0.01*.

**Table 11:** Regression model of the CCPT measures as the predictors of the CBCL social problem subscale score in the control group.

**Variable**	**Unstandardized B**	**Coefficient** **standard error**	**Standardized** **coefficient beta**	***t***	**Sig**.	***R***	***R*^**2**^**	**Adjusted *R*^**2**^**	***R*^**2**^ change**
**Step 1**									
SWAN ADHD score	−0.069	0.019	−0.475	−3.63	0.001	0.475	0.226	0.209	0.226
**Step 2**									
SWAN ADHD score	−0.065	0.018	−0.446	−3.55	0.001	0.559	0.313	0.282	0.087^*^
Commission	0.051	0.022	0.296	2.357	0.023				

*CCPT, Cued continued performance test; CBCL, Child behavior checklist; ADHD, Attention-deficit hyperactivity disorder; SWAN, the Strength and Weaknesses of ADHD Symptoms and Normal Behavior scale; Commission, CCPT commission errors*.

* *p < 0.05*.

### Use of AST Measures to Explain the CBCL Withdrawn and Social Problem Subscale Scores

A stepwise regression was performed again to determine the associations between the AST measures and the CBCL Withdrawn and Social Problem subscale scores. Again, the SWAN ADHD score was entered into the model first (Step 1), followed by the AST total correct and the AST reaction latency (mean) in a stepwise manner (Step 2). Bonferroni correction was used to adjust the *p*-value. The adjusted *p*-value was 0.0167 (0.05/3).

After controlling for the SWAN ADHD score, reaction latency (mean) was found to be the only significant independent variable that could explain the CBCL Social Problem subscale score in the high SCT group (Table 12). No other AST measure was a significant variable to explain the CBCL Social Problem or Withdrawn subscale score in either of the high SCT and control groups. Reaction latency (mean) alone explained 16.2% of the variance in the CBCL Social Problem subscale score, *F*_(1, 36)_ = 7.071, *p* < 0.0167, in the high SCT group. This result suggests that a faster response time in correct responses was negatively associated with more social problems among children with high SCT symptoms. Such results are contrary to the expectation that poorer performance in the selective attention task (i.e., a slower response time during correct responses) would be associated with more social problems. A possible explanation for the good performance of children with high SCT symptoms in the selective attention task may be that their anxiety improved their performance. Previous studies have shown that anxiety could enhance the inhibitory performance of individuals (30–32). Therefore, this explanation is plausible as children with high SCT symptoms are often reported to have higher anxiety problems. Furthermore, in the current study, the good performance of children with high SCT symptoms in the AST may be driven by anxiety, which may be associated with more social problems in real life. To support this explanation, further regression analysis was performed to examine whether anxiety problems were a mediator between AST reaction latency and social problems in the high SCT group.

**Table 12:** Regression model of the AST measures as the predictors of the CBCL social problem subscale score in the SCT group.

**Variable**	**Unstandardized B**	**Coefficient** **standard error**	**Standardized** **coefficient beta**	***t***	**Sig**.	***R***	***R*^**2**^**	**Adjusted *R*^**2**^**	***R*^**2**^ change**
**Step 1**									
SWAN ADHD score	−0.022	0.033	−0.108	−0.664	0.511	0.108	0.012	−0.015	0.012
**Step 2**									
SWAN ADHD score	−0.011	0.030	−0.054	−0.355	0.725	0.417	0.174	0.128	0.162^*^
Reaction latency (mean)	−0.005	0.002	−0.406	−2.66	0.012				

*AST, Attention switching task; CBCL, Child behavior checklist; SCT, Sluggish cognitive tempo; ADHD, Attention-deficit hyperactivity disorder; SWAN, the Strength and Weaknesses of ADHD Symptoms and Normal Behavior scale; Reaction latency (mean), mean of the AST reaction latency*.

* *p < 0.05*.

### Use of CBCL Anxiety/Depression Subscale Scores and AST Reaction Latency (Mean) to Explain the CBCL Social Problem Subscale Score in the High SCT and Control Groups

A linear regression was performed to examine how much variance of the CBCL Social Problem subscale score could be explained by the CBCL Anxiety/Depression subscales score and AST reaction latency (mean) individually. Again, the SWAN ADHD score was entered into the regression model first (Step 1), followed by the AST reaction latency (mean) (Step 2), and finally the CBCL Anxiety/Depression subscale score (Step 3). After controlling for the SWAN ADHD score, both the AST reaction latency (mean) and the CBCL Anxiety/Depression subscale score were found to be significant independent variables to explain the CBCL Social Problem subscale score in the high SCT group (Table 13). The CBCL Anxiety/Depression subscale score alone explained 25.3% of the variance in the CBCL Social Problem subscale score, whereas the AST reaction latency (mean) alone explained 16.3% of the variance in the CBCL Social Problem subscale score in the high SCT group. In the control group, neither the AST reaction latency (mean) nor the CBCL Anxiety/Depression subscale score was a significant independent variable to explain the CBCL Social Problem subscale score (Table 14).

**Table 13:** Regression model of the AST reaction latency and the CBCL Anxiety/Depression subscale scores as the predictors of the CBCL social problem subscale score in the SCT group.

**Variable**	**Unstandardized B**	**Coefficient** **standard error**	**Standardized** **coefficient beta**	***t***	**Sig**.	***R***	***R*^**2**^**	**Adjusted *R*^**2**^**	***R*^**2**^ change**
**Step 1**									
SWAN ADHD score	−0.023	0.034	−0.116	−0.691	0.494	0.116	0.013	−0.015	0.013
**Step 2**									
SWAN ADHD score	−0.012	0.031	−0.062	−0.393	0.696	0.420	0.176	0.128	0.163^*^
Reaction latency (mean)	−0.005	0.002	−0.407	−2.59	0.014				
**Step 3**									
SWAN ADHD scoreReaction latency (mean)Anxiety_Depressed	0.006 −0.005 0.291	0.027 0.002 0.076	0.028 −0.392 0.511	0.206 −2.95 3.821	0.838 0.006 0.001	0.655	0.429	0.377	0.253^**^

*AST, Attention switching task; CBCL, Child behavior checklist; SCT, Sluggish cognitive tempo; ADHD, Attention-deficit hyperactivity disorder; SWAN, the Strength and Weaknesses of ADHD Symptoms and Normal Behavior scale; Reaction latency (mean), mean of the AST reaction latency; Anxiety_Depressed, CBCL Anxiety/Depressed subscale raw score*.

* *p < 0.05*,

** *p < 0.01*.

**Table 14:** Regression model of the AST reaction latency and the CBCL Anxiety/Depression subscale scores as the predictors of the CBCL social problem subscale score in the control group.

**Variable**	**Unstandardized B**	**Coefficient** **standard error**	**Standardized** **coefficient beta**	***t***	**Sig**.	***R***	***R*^**2**^**	**Adjusted *R*^**2**^**	***R*^**2**^ change**
**Step 1**									
SWAN ADHD score	−0.069	0.019	−0.475	−3.59	0.001	0.475	0.226	0.209	0.226^**^
**Step 2**									
SWAN ADHD score	−0.069	0.020	−0.475	−3.55	0.001	0.476	0.227	0.191	0.001
Reaction latency (mean)	0.000	0.002	0.028	0.216	0.830				
**Step 3**									
SWAN ADHD score Reaction latency (mean) Anxiety_Depressed	−0.062 0.000 0.174	0.019 0.002 0.073	−0.426 0.028 0.312	−3.30 0.216 2.376	0.002 0.830 0.022	0.564	0.318	0.270	0.092^*^

*AST, Attention switching task; CBCL, Child behavior checklist; ADHD, Attention-deficit hyperactivity disorder; SWAN, the Strength and Weaknesses of ADHD Symptoms and Normal Behavior scale; Reaction latency (mean), mean of the AST reaction latency; Anxiety_Depressed, CBCL Anxiety/Depressed subscale raw score*.

* *p < 0.05*,

** *p < 0.01*.

## Discussion

The aim of this study was to examine the associations between sustained/selective attention and social problems/withdrawn behavior in children with SCT. The results demonstrated that sustained attention, as measured by the CCPT omission errors, was a significant variable to explain social problems and withdrawn behavior in children with high SCT symptoms after controlling for ADHD symptoms. Selective attention, as measured by the AST reaction latency (mean), was also identified as a significant variable that could explain social problems in children with SCT. Thus, these results suggest the presence of a link between sustained attention difficulties and social problems/withdrawn behavior in children with SCT. Notably, faster response time in the AST was found to be the only significant variable that could explain social problems in children with SCT.

### Behavioral Difference Between Children With SCT and Those With Typical Development

The results of this study showed that children in the high SCT group had significantly more withdrawn behavior, anxiety/depression, and attention and social problems than those in the control group. This is consistent with previous studies (12, 33) that found that SCT symptoms were associated with social problems, anxiety, depression, and withdrawn behavior in children in the U.S. (1). The results of our study confirm that these impairments are characteristic in children with SCT.

### Sustained Attention Difficulties and Social Problems/Withdrawn Behavior in SCT

Previous studies have shown that SCT symptoms are more related to sustained attention difficulties than executive function deficits (7, 18, 19). However, no study has yet examined the link between such attention difficulties and the unique withdrawn behavior/social problems in children with SCT. Our results demonstrated that even after controlling for ADHD symptoms, omission errors in the CCPT were a significant variable to explain social problems and withdrawn behavior in the high SCT group. In the control group, withdrawn behavior and social problems could be significantly explained by commission errors in the CCPT. Therefore, social problems/withdrawn behavior were more related to sustained attention difficulties in children with SCT, while more related to impulsivity in children with typical development. The severity of SCT symptoms has been found to be associated with the neurophysiological state of arousal (34), which in turn may cause sustained attention deficit among these children (35). Sustained attention deficit may negatively affect the ability of these children to remain vigilant and attend to important social cues during social conversations and interactions (16, 17). Given such attention deficits, it is not surprising that children with SCT exhibited passive and withdrawn behavior during their interaction with others in the present study.

### Selective Attention Difficulties and Social Problems/Withdrawn Behavior in SCT

Selective attention has been suggested to be related to social behavior. It enables an individual to attend to relevant social information while suppressing the non-relevant information in the encoding stage of social information processing (36–38). Children with SCT have been found to exhibit more deficits in selective attention processing than children without SCT (21) and children with only ADHD (19). Therefore, it was expected that selective attention deficit would predict social problems and withdrawn behavior in children with high SCT in the current study. However, we found that the AST reaction latency (mean) was the only variable that could explain social problems in the high SCT group after controlling for ADHD symptoms. The AST total correct was found to be significantly and negatively correlated with both social problems and withdrawn behavior in the high SCT group only. However, it was a non-significant variable to explain social problems and withdrawn behavior after controlling for ADHD symptoms. In the present study, the AST reaction latency (mean) was found to be negatively correlated with social problems even after controlling for ADHD symptoms. This result suggests that children in the high SCT group who had a faster response time (shorter reaction latency) in the AST tended to have more social problems in their real life. This result may be explained by a previous report that anxiety likely improves the performance of individuals in tasks that require inhibitory control, especially of individuals who have weak attentional control (31, 32). Specifically, individuals with weak attentional control may benefit from increased arousal during a challenging task, as it can help them to better cope with the inhibitory nature of the task (31). In contrast, individuals with high attentional control could perform consistently well no matter which testing environment they are in. Therefore, the fast reaction time in the AST may reflect the underlying difficulties in attentional control among children with SCT. A previous study also found that higher SCT symptoms were associated with faster speed in tasks requiring inhibitory control (39). The authors interpreted these results to indicate that individuals with SCT may have an overactive behavioral inhibition system, which overinhibited their behavior. Subsequently, they may appear inactive or avoidant in the social world. Therefore, it was not surprising that faster reaction time in the AST was found to be associated with more social problems in the current study.

The CBCL Anxiety subscale was not found to be a mediator between the AST reaction latency and the CBCL Social Problem subscale in the additional regression analysis; both the AST reaction latency and CBCL Anxiety subscale were significant independent factors to explain social problems among children with high SCT symptoms. These results suggest that social problems in SCT may be related to two independent factors: the general anxiety level measured by the CBCL and the attentional control difficulties reflected by the faster reaction time during the AST. Overall, the results of the present study suggest that the cognitive processes of sustained attention and attentional control are related to social difficulties frequently reported by children with high SCT symptoms.

### Significance of the Study

This study is the first to investigate the neuropsychological correlates of social problems and withdrawn behavior, which are hallmark features of high SCT, among children with high SCT. However, not much is known about the underlying neuropsychological factors that contribute to these clinical features of SCT. The results of this study support the presence of a link between sustained attention difficulty (reflected by omission errors) and social problems/withdrawn behavior as well as a link between attentional control difficulty (reflected by fast reaction time in the AST) and social problems in children with high SCT symptoms. Therefore, the clinical features of SCT (such as social problems and withdrawn behavior) may be the result of deficits in sustained attention and attentional control abilities. The current results may help to build a theoretical explanation for the social problem in children with SCT.

Besides providing further understanding about the nature of social difficulties in SCT, our results provide possible insights into the symptomology and associated problems in other developmental disorders. For example, a previous study reported that SCT symptoms, but not ADHD symptoms, were positively associated with autism symptoms such as weak reciprocal social interaction (40). In addition, another study demonstrated that compared with individuals with ASD symptoms and low SCT symptoms, those with ASD symptoms and high SCT symptoms had significantly higher social impairments (41). Likewise, the results of the current study provide a plausible new perspective in understanding the social problems experienced by children with autism. For example, the present study found that sustained attention and selective attention difficulties were related to social problems and withdrawn behavior among children with high SCT symptoms, while a previous study reported that high SCT symptoms were related to social problems in children with autism (41). Thus, the social difficulties experienced by children with autism may also be explained by sustained attention and selective attention deficits. Further studies are needed to examine the relationship between sustained/selective attention deficits, SCT symptoms, and social problems in children with ASD.

### Limitations of the Study

This study had some limitations worth noting. Firstly, the sample size was small in the current study. Therefore, more studies with larger sample size are needed in order to confirm the findings of the present study. Secondly, although the results of this study suggest a link between sustained attention/attentional control and social problems in children with SCT, it is not clear which aspects of the social problem (e.g., peer rejection or initiation to communication) or which stage of social information processing (e.g., encoding or response selection) these attention difficulties are related to. Future studies should further investigate the nature of social problems and/or deficits in social information processing in children with SCT and how the above-mentioned attention deficits are related to different aspects of social problems and social information processing. One approach is to measure the electroencephalography (EEG) of the individuals with SCT while they are performing a simulated social task [e.g., simulated chat room task; (16)]. Examination of the individual EEG frequency bands may clarify the attentional states of these individuals in different social situations during the task. Such evidence may confirm the causal link between attention deficits and social problems in these individuals.

## Conclusion

This study demonstrated that sustained attention and attentional control are the two attentional processes that may explain the social problems and withdrawn behavior in children with high SCT symptoms. This suggests that attention deficits are among the core neuropsychological deficits that can account for the social problems experienced by children with SCT. This understanding of the relationship between the neuropsychological deficits in SCT and their associated problems may inform the development of a more comprehensive model of SCT and of better treatment approaches for individuals with this condition.

## Data Availability Statement

The raw data supporting the conclusions of this article will be made available by the authors, without undue reservation.

## Ethics Statement

The studies involving human participants were reviewed and approved by Human Ethics Committee, Department of Rehabilitation Sciences, The Hong Kong Polytechnic University. Written informed consent to participate in this study was provided by the participants' legal guardian/next of kin.

## Author Contributions

TY and CL were responsible for the research design, data collection, writing of manuscript, and final editing. SN, CC, and JC were responsible for reviewing the manuscript and final editing. All authors contributed to the article and approved the submitted version.

## Conflict of Interest

The authors declare that the research was conducted in the absence of any commercial or financial relationships that could be construed as a potential conflict of interest.
